# 4-month omalizumab efficacy outcomes for severe allergic asthma: the Dutch National Omalizumab in Asthma Registry

**DOI:** 10.1186/s13223-017-0206-9

**Published:** 2017-07-26

**Authors:** S. M. Snelder, E. J. M. Weersink, G. J. Braunstahl

**Affiliations:** 1 Franciscus Gasthuis & Vlietland, Kleiweg 500, 3045 PM Rotterdam, The Netherlands; 2 Academisch Medisch Centrum, Amsterdam, The Netherlands

**Keywords:** Allergic asthma, Omalizumab, ACQ, FEV1

## Abstract

**Background:**

Omalizumab is licensed as add-on therapy for patients with severe allergic asthma. Response is in most studies scored by the physician’s global evaluation of treatment effectiveness (GETE). A good clinical and validated parameter for treatment response is currently missing. Also, there are no established criteria for identifying patients who will respond to omalizumab based on pre-treatment characteristics. The Dutch National Omalizumab in Asthma Registry was developed in 2011 to better evaluate inclusion criteria and measure treatment response after 4 months.

**Methods:**

This is a “real world” prospectively designed, observational data registry in which the outcomes of patients who received omalizumab between 2012 and 2015 were evaluated. Data were collected from all centers in the Netherlands comprising demographic features, criteria for starting treatment, GETE, FEV1, oral corticosteroid use and ACQ.

**Results:**

65.5% of the 403 patients had a good or excellent response to omalizumab after 16 weeks according to the treating physician GETE. 64.5% fulfilled all the criteria for prescribing omalizumab at baseline. The mean ACQ improved from 2.96 at baseline to 1.83 at 16 weeks (p < 0.001). 75.3% of the responders showed more than 0.5 points improvement in the ACQ. The mean FEV1 increased from 71.58 to 79.06 (p < 0.001). There was no relationship between patients with a FEV1 <80 and ≥80% at baseline and response (p = 0.981). Most of the responders had a considerable improvement of FEV1 either/or ACQ or OCS use (88.3%). While 86.7% of the responders had an improvement of either ACQ or FEV1. 75.4% of the responders had an improvement of ACQ, while 50.4% had an improvement of FEV1. Finally 11.7% of the patients with no improvement of FEV1, ACQ or OCS use were considered to have a good response.

**Conclusions:**

This registry of 403 inadequately controlled severe allergic asthma patients in the Netherlands showed a good or excellent response of 65.5% to omalizumab after 16 weeks, in accordance with previous studies. The assumption that careful registration would lead to higher response rates could not be supported by the data from this registry. Improvement of ACQ appears to be a useful additional assessment tool to measure response in omalizumab treated patients.

## Background

Omalizumab (Xolair^®^) is a subcutaneously administrated humanized anti-immunoglobulin E (IgE) monoclonal antibody that targets circulating free IgE and prevents its interaction with the high-affinity IgE receptor (FCƐR1). It is licensed in the European Union as add-on therapy for patients aged 6 years and older with either allergic asthma or chronic idiopatic urticaria [1, 2]. Since 2006, omalizumab has been prescribed for inadequately controlled severe allergic asthma in the Netherlands. Randomized studies demonstrated a significantly greater improvement in asthma control in patients treated with add-on omalizumab than patients treated with placebo [3–6].

Response is in most studies scored by the physician’s global evaluation of treatment effectiveness (GETE). The physicians GETE is a composite measure that encompasses multiple aspects of evaluation of response, including patient interviews, review of medical notes, spirometry and diaries of symptoms and rescue medication [7]. As GETE is a subjective parameter for response we want to search for a more objective parameter. Other often used measurements for improvement are the asthma control questionnaire (ACQ) [8], asthma control test (ACT) [9], asthma quality-of-life questionnaire (AQLQ) [10], mini-AQLQ [11], asthma symptom score, FEV1 and exacerbation rate [3, 4, 12]. A single good parameter for response is missing.

EU indication for prescribing omalizumab is: severe persistent (IgE-mediated) allergic asthma, positive skin test or in vitro reactivity to a perennial aeroallergen, frequent daytime symptoms or night-time awakenings, multiple documented severe asthma exacerbations despite daily high-dose ICS plus a LABA and in patients >12 years reduces lung function (FEV1 <80%) [1]. Criteria for prescribing omalizumab in Australia are the same as in the EU, except that the FEV1 had to be documented less than 80% on more than three occasions in the previous 3 months [13]. In the USA a FEV1 <80% is not a criteria for prescribing [14].

At present, there are no established criteria for identifying patients who will respond to omalizumab based on pre-treatment characteristics [15]. Initially, omalizumab was started in some patients that did not strictly fulfill the criteria for omalizumab prescription [12]. In 2011, the Dutch reimbursement authority required more data about starting criteria and treatment response which lead to the formation of Dutch National Omalizumab in Asthma Registry. The organization and monitoring was in the hands of the Dutch Organization of Chest Physicians (NVALT). The assumption was that a stricter registration policy would lead to higher response rates and therefore be more cost-effective. Moreover, several clinical parameters were monitored to see which ones would best objectively relate to treatment response.

## Methods

This is a “real world” prospectively designed, observational data registry in which the outcomes of patients who received omalizumab between 2012 and 2015 were evaluated. Data were collected from all centers in the Netherlands where omalizumab was prescribed for the treatment of severe allergic asthma. The survey questionnaire was approved by the national board of Chest Physicians (NVALT) and comprised the following start criteria: severe allergic asthma, age >6 years, a positive skin test or in vitro activity to a relevant perennial aeroallergen, a FEV1 less than 80%, more than two severe exacerbations and substantial symptoms despite treatment with inhaled corticosteroids (ICS) and long-acting B2-agonists (LABAs). In addition, inhalation technique and compliance were checked and optimized, and smoking stopped (or at least tried). Patients gave informed consent to participate in the survey. The data were centrally collected and analyzed by three independent physicians.

### Response evaluation

Response was defined as a physician-rating GETE of excellent or good. Non-response was defined as a physician-rating GETE of moderate, poor or worsening. Response evaluation was left to the discretion of the treating physician. However, it was strongly recommended to measure ACQ-6 and FEV1 at baseline, at 2 and 4 months. Also, when patients were on maintenance therapy with oral corticosteroids the average daily dose was registered. The ACQ-6 includes both patient-reported symptoms and use of rescue medication [8]. ACQ scores range from 0 (completely controlled) to 6 (extremely poorly controlled). A decrease in ACQ score of more than 0.5 points is considered to be the minimal clinically important improvement [11].

A sub-analysis was performed between patients with FEV1 ≥80% and FEV1 <80% at baseline.

### Statistical analysis

The unpaired Student’s *t* test was used for continuous variables with normal distributions, and Chi square test/Fisher’s Exact test for categorical variables. A p value <0.05 (two-sided) is considered a statistically significant difference. Correlation was measured using the Spearman Rank correlation coefficient. Statistical analyses were performed using SPSS version 18.0 (SPSS Inc., Chicago, Illinois, USA).

## Results

403 patients had a full data set and could be evaluated. Baseline characteristics are shown in Table 1. The mean age was 47. 62.8% of the patients were female. The mean IgE was 619.9 with a range from 3 to 10,800. 69.2% had a FEV1 <80% of predicted. 64.5% of the patients fulfilled all of the criteria for prescribing omalizumab at baseline (Table 2).

**Table 1:** Baseline characteristics

Variable	Value
Total no patients	403
Age	
Mean (SD)	47 (15.6)
Gender (%)	
Male	149 (37.0)
Female	253 (62.8)
Body weight, kg	
Mean (SD)	78.6 (17.4)
Baseline IgE level, IU/mL	
Mean (SD)	619. 9 (1036.4)
Range	3–10,800
Severe allergic asthma (%)	
Yes	380 (94.3)
No	12 (3.0)
Positive skin-prick test/RAST (%)	
Yes	363 (90.3)
No	26 (6.5)
FEV1 <80 (%)	
Yes	279 (69.2)
No	116 (28.8)
More than 2 exacerbations (%)	
Yes	384 (95.3)
No	11 (2.7)
Maximum dose ICS and LABAs (%)	
Yes	394 (99)
No	4 (1)
Smoking (%)	
Tried to quit smoking	101 (25.1)
Didn’t try to quit smoking	29 (7.2)

**Table 2:** Fulfilled all the criteria for prescribing omalizumab at baseline vs response

	Response		
	Yes	No	Total
Criteria fulfilled yes	173	87	260
Criteria fulfilled no	91	52	143
Total	264	139	403
	*p* *=* *0.558*		

65.5% of the patients had a good or excellent response to omalizumab after 16 weeks according to the treating physician GETE. Table 3 shows if the patients fullfilled the criteria for prescribing omalizumab and if they had good response to omalizumab. As shown in Table 4, the mean ACQ improved from 2.96 at baseline to 1.83 at 16 weeks (p < 0.001). 75.3% of the responders showed more than 0.5 points improvement in the ACQ score after 16 weeks. The mean FEV1 increased from 71.58 to 79.06 (p < 0.001). 50.4% of the responders had an improvement of ≥5% of FEV1 %pred. There was no relation between FEV1 <80%pred and ≥80%pred at baseline and response after 4 months (p = 0.981). Table 4 shows that the maintenance OCS use was lower at 16 weeks (66.5% none at baseline vs 72.2% none at 16 weeks) p < 0.001. The response remained stable over the years 2012–2015, p = 0.690.

**Table 3:** Criteria for prescribing omalizumab fulfilled and good response

	Fulfilled/good response	Not fulfilled/good response
Severe allergic asthma	380/250	12/5
Age >6 years	403/263	0/0
A positive skin test or RAST	364/241	26/12
FEV1 <80	279/183	116/77
>2 exacerbations	384/250	11/7
Maximum dose LABAs and ICS	394/257	4/3

**Table 4:** ACQ, FEV1 and OCS at baseline vs 16 weeks

	Baseline	16 weeks
ACQ		
n	334	307
Mean (SD)	2.96 (1.12)	1.83 (1.12)
		*p* < *0.001*
FEV1		
n	338	287
Mean (SD)	71.58 (19.6)	79.06 (20.06)
		*p* < *0.001*
OCS		
n	364	334
None (%)	242 (66.5)	241 (72.2)
1–5 mg (%)	45 (12.4)	46 (13.8)
6–9 mg (%)	15 (4.1)	12 (3.6)
>10 mg (%)	61 (16.8)	35 (10.5)
		*p* *<* *0.001*

**Fig. 1:**
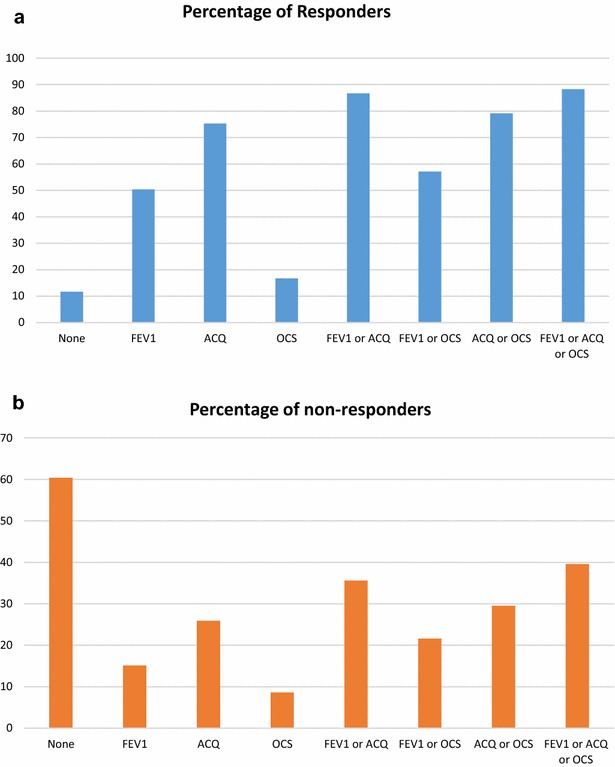
**a** Percentage of responders versus an improvement of FEV1, ACQ or OCS. **b** Percentage of non-responders versus an improvement of FEV1, ACQ or OCS

Figure 1a shows that most of the responders had an improvement of either FEV1 or ACQ or OCS (88.6%). While 86.7% of the responders had an improvement of either FEV1 or ACQ. It also shows that ACQ alone (75.4%) appears to be a better measurement for a response than either improvement of the FEV1 (50.4%) or OCS use (16.7%). Figure 1b shows that 60.4% of the non-responders had neither an improvement of ACQ nor FEV1 nor OCS. 66.5% of the patients who fulfilled all of the criteria at baseline had a good or excellent response (Table 2). There is no relationship between fulfilling all the criteria and response (p = 0.558).

11.7% of the patients with no improvement of the ACQ, FEV1 or OCS had a good response. There was a sufficient degree of correlation between improvement of ACQ and response according to GETE [r = 0.458, p < 0.001(=7.92E−18)] and a weak correlation between improvement of FEV1 and response according to GETE [r = 0.292, p < 0.001(=4.98E−7)].

## Discussion

65.5% of the 403 patients with inadequately controlled severe allergic asthma had a good or excellent response to omalizumab after 16 weeks. 75.3% of the responders had more than 0.5 points improvement of the ACQ. Overall the ACQ improved, FEV1 increased and there was lower use of OCS at 16 weeks. 50.4% of the responders had an improvement of more than 5% of the FEV1. More patients who had a good or excellent response had an improvement of the ACQ (75.3%) than an improvement of FEV1 (50.4%) or OCS use (16.7%). This suggests that the ACQ may be the best measurement for response.

We found a response rate of 65.5%, which is in accordance with previous data from randomized controlled trials and real world data. Bousquet et al. found a response of 62.0% at 16 weeks [3]. Niven et al. found a response of 70% [5]. Schumann et al. had a response of 78.8% in their prospective multicenter study [16]. Brusselle et al. even showed that more than 82% had good/excellent GETE [17]. The eXpeRience registry showed a response of 69.9% after 16 weeks by GETE [12]. The assumption that careful registration would lead to higher response rates could not be proven in this study. In fact, only 64.5% fulfilled all of the criteria for prescribing omalizumab at baseline. There is no relationship between fulfilling all the criteria and response (p = 0.558). The main reason for not strictly following the rules was a FEV1 >80 (28.8% of the total population), followed by missing of a positive skin test or in vitro activity to a perennial aeroallergen (Table 1).

Schumann et al. described that the ACQ score significantly decreased from 3.58 ± 1.28 to 2.01 ± 1.05 after 16 weeks (−43.7%), treatment responders showed greater and highly significant improvements of symptoms compared with non-responders even after 16 weeks (−46.9%, p < 0.0001 vs −36.1%, p < 0.05) [16]. This is in agreement with our findings and underlines the importance of using ACQ in response evaluation. According to FEV1 they described that patients who did respond to omalizumab treatment had higher absolute FEV1 values at baseline (2.11 L vs 1.87 L) and showed a higher expressed increase in % predicted of FEV1 compared with non-responders (15.6% vs 13.7%) [16]. We didn’t find a relationship between patients with a FEV1 <80 and ≥80% at baseline and response (p = 0.981).

There was a significant lowering of OCS use at 16 weeks, these results are in line with the eXpeRience registry [18]. The evaluation period of 4 months was too short to say something about exacerbations.

Despite our positive findings, it is important to recognize the limitations of our study. First as with all observational studies, is the lack of a control group and the open-label design. Other limitations of observational studies are that the results should be interpreted with due consideration that factors other than the treatment of interest may have contributed to the findings. Finally, the data quality relied heavily on the accuracy and completeness of available clinical records.

## Conclusions

This registry of 403 inadequately controlled severe allergic asthma patients in the Netherlands showed a good or excellent response of 65.5% to omalizumab after 16 weeks. Overall the ACQ improved, FEV1 increased and there was lower use of OCS at 16 weeks. This is in accordance with previous data from randomized controlled trials and real word data. 75.3% of the responders had more than 0.5 points improvement of the ACQ. There was no relationship between patients with a FEV1 <80 and ≥80% at baseline and the response. Improvement of ACQ appears to be a useful assessment tool to measure response in omalizumab treated patients.

## Abbreviations

GETE: global evaluation of treatment effectiveness
ACQ: asthma control questionnaire
FEV1: forced expiratory volume in 1 s
ACT: asthma control test
AQLQ: asthma quality-of-life questionnaire
